# DirectMX – One-Step Reconstitution of Membrane Proteins From Crude Cell Membranes Into Salipro Nanoparticles

**DOI:** 10.3389/fbioe.2020.00215

**Published:** 2020-03-19

**Authors:** Pilar Lloris-Garcerá, Stefan Klinter, Liuhong Chen, Michael J. Skynner, Robin Löving, Jens Frauenfeld

**Affiliations:** ^1^Salipro Biotech AB, Stockholm, Sweden; ^2^Bicycle Therapeutics, Cambridge, United Kingdom

**Keywords:** Salipro nanoparticles, direct membrane extraction, DirectMX, GPCR, SLC transporter, membrane protein, drug discovery, saposin

## Abstract

Integral membrane proteins (IMPs) are central to many physiological processes and represent ∼60% of current drug targets. An intricate interplay with the lipid molecules in the cell membrane is known to influence the stability, structure and function of IMPs. Detergents are commonly used to solubilize and extract IMPs from cell membranes. However, due to the loss of the lipid environment, IMPs usually tend to be unstable and lose function in the continuous presence of detergent. To overcome this problem, various technologies have been developed, including protein engineering by mutagenesis to improve IMP stability, as well as methods to reconstitute IMPs into detergent-free entities, such as nanodiscs based on apolipoprotein A or its membrane scaffold protein (MSP) derivatives, amphipols, and styrene-maleic acid copolymer-lipid particles (SMALPs). Although significant progress has been made in this field, working with inherently unstable human IMP targets (e.g., GPCRs, ion channels and transporters) remains a challenging task. Here, we present a novel methodology, termed DirectMX (for direct membrane extraction), taking advantage of the saposin-lipoprotein (Salipro) nanoparticle technology to reconstitute fragile IMPs directly from human crude cell membranes. We demonstrate the applicability of the DirectMX methodology by the reconstitution of a human solute carrier transporter and a wild-type GPCR belonging to the human chemokine receptor (CKR) family. We envision that DirectMX bears the potential to enable studies of IMPs that so far remained inaccessible to other solubilization, stabilization or reconstitution methods.

## Introduction

Integral membrane proteins (IMPs) represent ∼23% of the human proteome (Uhlén et al., 2015), and carry out key functions in many essential cellular processes including signal transduction, membrane trafficking, cellular metabolism, and nutrient transport (Bolla et al., 2019). Different classes of IMPs such as G protein-coupled receptors (GPCRs), ion channels and transporters are of great interest for the pharmaceutical industry as they represent targets for more than 60% of the drugs in clinical use (Overington et al., 2006; Rask-Andersen et al., 2011). IMPs are anchored in the cell membrane where they interact with a dynamic and complex lipid environment, known to be important for the stability and function of the proteins (Whorton and MacKinnon, 2011; Contreras et al., 2012; Laganowsky et al., 2014; reviewed by Hedger and Sansom, 2016; Pyle et al., 2018). Traditionally, detergents are used to extract IMPs from cell membranes. Although providing an amphiphilic micelle environment, detergents are poor mimics of the cell membrane lipids as they lack major structural features of lipid molecules (i.e., charged headgroup, specific acyl chains), which are important to keep solubilized IMPs stable and functional (Zoonens et al., 2013; Alvarez et al., 2015; Martens et al., 2016; Nji et al., 2018). In addition, the continuous presence of detergents leads to delipidation of IMPs and may negatively affect the experimental setup and downstream processes.

To overcome IMP instability in detergent, protein engineering by introducing thermostabilising mutations has been a successful approach, especially in the field of structural biology. However, protein engineering is time-consuming and leads to IMP stabilization in a preferred conformational state not revealing the full structural and functional complexity of the native IMP (reviewed by Tate, 2012; Magnani et al., 2016). In addition, several technologies have been developed in order to study IMPs in a detergent-free environment, including proteoliposomes (Rigaud et al., 1995), virus-like particle (VLP) systems (Willis et al., 2008), amphipols (Tribet et al., 1996), nanodiscs (Bayburt and Sligar, 2003; reviewed by Denisov and Sligar, 2017), and SMALPs (Knowles et al., 2009; Lee et al., 2016). While all the listed technologies may be suitable for a given IMP and application, it remains challenging to extract and purify wild-type human IMPs, thus hampering research and drug discovery efforts.

We have recently developed a novel technology for the reconstitution of detergent-purified IMPs into Salipro nanoparticles (Frauenfeld et al., 2016). Here, the lipid-binding protein SapA forms a scaffold that surrounds disk-like nanoparticles comprising lipids and IMPs. The advantage of the Salipro technology is that the SapA scaffold flexibly adjusts to the size of the incorporated IMP, thereby facilitating the incorporation of IMPs of various sizes and structures (Frauenfeld et al., 2016; Flayhan et al., 2018; Nguyen et al., 2018). Salipro nanoparticles are highly homogeneous, displaying both increased thermostability and half-life. In addition, Salipro-reconstituted IMPs have been shown to be functional and structurally intact by a wide range of biochemical and biophysical methods such as cryogenic EM, nuclear magnetic resonance spectroscopy or enzymatic assays (Frauenfeld et al., 2016; Chien et al., 2017; Flayhan et al., 2018; Kintzer et al., 2018; Nguyen et al., 2018; Kanonenberg et al., 2019; Kehlenbeck et al., 2019; Nagamura et al., 2019).

**FIGURE 1:**
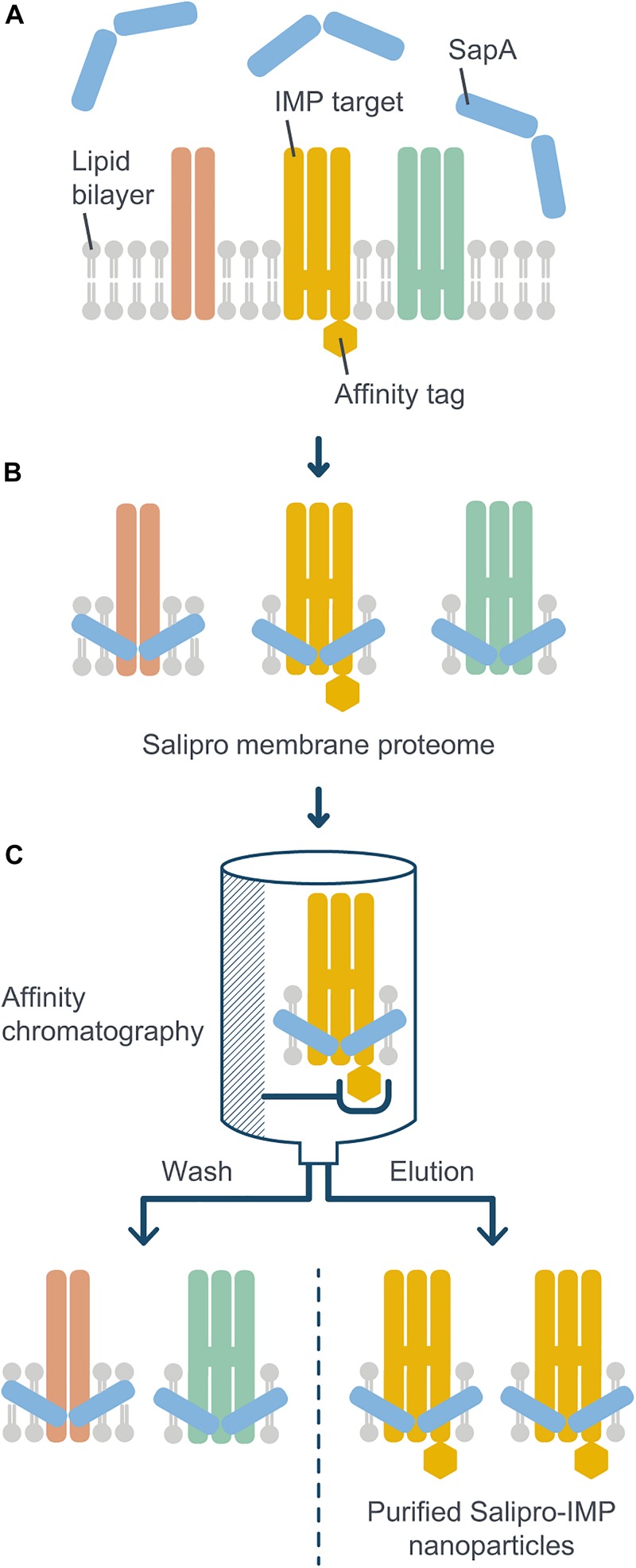
Schematic illustration of the DirectMX methodology. The reconstitution of IMPs directly from crude cell membranes into Salipro nanoparticles is shown here for a recombinantly expressed example protein. **(A)** For DirectMX reconstitution of IMPs, homogenized crude cell membranes are incubated with digitonin and SapA. **(B)** The lipid-binding ability of SapA allows for self-assembly of native membrane lipid disks, thereby reconstituting IMPs into a scaffold of SapA molecules. **(C)** If the target IMP is fused to an affinity tag, the protein-containing nanoparticles can be readily purified by affinity chromatography using detergent-free buffers. The purified nanoparticles are then available for further downstream processes, such as SEC, or any kind of biochemical, structural or biophysical analysis.

As mentioned above, many IMPs are difficult to study because of the lack of suitable extraction and purification protocols. Solving this fundamental scientific problem has the potential to facilitate research and drug discovery efforts against these therapeutically important proteins. To address this challenge, we present a novel Salipro methodology, termed DirectMX (for direct membrane extraction), which allows for a rapid one-step reconstitution of IMPs from crude cell membranes into Salipro nanoparticles (Figure 1). DirectMX uses the endogenous lipids present in a crude cell membrane preparation to first reconstitute all IMPs of the sample into a population of stable Salipro nanoparticles. Subsequently, the Salipro nanoparticles carrying the IMP of interest are readily purified by affinity chromatography using an affinity tag engineered to the target protein. In this proof-of-concept study, we illustrate the feasibility of the DirectMX workflow by the reconstitution of two IMPs: A human solute carrier family 1 (SLC) transporter and a wild-type human GPCR belonging to the CKR family, which were both recombinantly expressed in human cells.

## Materials and Methods

### Materials and Reagents

Human SapA was recombinantly produced and purified as described previously (Frauenfeld et al., 2016; Lyons et al., 2017). Pure SapA in HNG buffer (50 mM HEPES at pH 7.5, 150 mM NaCl, 5% (v/v) glycerol) was stored at −80°C. A human SLC transporter was used as exemplary IMP, namely a version of the excitatory amino acid transporter 1 known as EAAT1_*cryst*_ (Canul-Tec et al., 2017). An EAAT1_*cryst*_ construct, engineered with an N-terminal Strep-tag II affinity tag followed by a daGFP reporter and a PreScission protease cleavage site, was recombinantly produced (Evotec) in transiently transfected HEK293 suspension cells. Crude membranes were isolated and homogenized (Evotec) according to Canul-Tec et al. (2017) in a buffer containing 50 mM HEPES (pH 7.4), 200 mM NaCl, 1 mM L-asparagine, 1 mM EDTA, 1 mM TCEP, and 10% (v/v) glycerol, and stored at −80°C. In addition, an undisclosed wild-type human CKR construct with N-terminal Myc and DDK affinity tags in tandem was recombinantly produced in transiently transfected HEK293S suspension cells. Crude membranes were isolated and homogenized in HNG buffer, and stored at −80°C. Highly pure digitonin (Merck) was prepared as 5% (w/v) stock and stored at −20°C. FLAG peptide (Sigma-Aldrich) was prepared as 5 g L^–1^ stock and stored at −20°C.

### DirectMX Reconstitution of SLC and CKR Into Salipro Nanoparticles

All steps were performed at 4°C. For DirectMX reconstitution of SLC from crude membranes, 500 μL of homogenized crude membranes (0.7 g L^–1^) were adjusted to 1 mL with HN buffer supplemented with digitonin (final concentrations of 50 mM HEPES at pH 7.5, 150 mM NaCl and 1% digitonin), and incubated on a rotator for 1 h. Insoluble material was removed by centrifugation at 30,000 × *g* for 45 min. Solubilized membrane material containing native lipids and IMPs was mixed with 16.5 mL of SapA (0.6 g L^–1^) in detergent-free HN buffer and incubated on a rotator for 30 min to allow the reconstitution of IMPs into Salipro nanoparticles. For CKR, 500 μL of homogenized crude membranes were mixed HNG buffer and digitonin (1% final concentration) in a total volume of 5 mL, and 8 mL of SapA (3 g L^–1^) were used instead.

### Chromatographic Purification of Salipro-SLC and Salipro-CKR Nanoparticles

All steps were performed at 4°C unless indicated otherwise and in detergent-free buffers. For affinity chromatography using the Strep-tag II affinity tag of SLC, ∼500 μL of StrepTactin Sepharose High Performance resin (GE Healthcare), equilibrated with 5 CVs of HN buffer, were added to the population of Salipro nanoparticles and incubated on a rotator for 1 h. The suspension was loaded onto an empty gravity flow column and the flow-through was passed once more over the resin. After washing the column with 10 CVs of HN buffer, Salipro-SLC nanoparticles were eluted with 4 CVs of HN buffer containing 2.5 mM D-desthiobiotin. For affinity chromatography using the DDK affinity tag of CKR, ∼500 μL of anti-FLAG M2 resin (Sigma-Aldrich), equilibrated with 5 CVs of HNG buffer, were used, washed with fifteen CVs of HNG buffer, and Salipro-CKR nanoparticles eluted twice with 5 CVs of HNG buffer containing FLAG peptide (0.25 g L^–1^). A sample of the eluates was used for analytical SEC and the rest concentrated using Amicon membrane-based ultrafiltration devices (Merck) with 100-kDa NMWL (SLC) or 30-kDa NMWL (CKR) at 3,000 × *g*. Protein samples of the different purification steps were analyzed by SDS-PAGE at room temperature using NuPAGE 4–12% Bis-Tris polyacrylamide gels (ThermoFisher Scientific) and either visualized by silver staining or used for Western blotting.

For analytical SEC, samples of 50 μL were loaded onto Superose 6 Increase 5/150 GL (SCL) or Superdex 200 Increase 5/150 (CKR) columns (GE Healthcare), equilibrated in HNG buffer, using a Prominence-i LC-2030C high performance liquid chromatography system equipped with PDA and RF-20Axs fluorescence detectors (Shimadzu) at a flow rate of 0.3 mL min^–1^. In-line light absorbances were monitored for total protein elution at 280 nm and for daGFP elution at 512 nm, respectively, with excitation for the latter set to 500 nm.

For subsequent fractionation of the concentrated eluate from the affinity chromatography step by preparative SEC, ∼300 μL of the eluate were loaded onto Superose 6 Increase 10/300 GL (SCL) or Superdex 200 Increase 10/300 (CKR) columns (GE Healthcare), equilibrated in HNG buffer, using an Äkta pure chromatography system (GE Healthcare) at a flow rate of 0.35 mL min^–1^. Protein fractions were analyzed by SDS-PAGE as described above. Fractions containing pure Salipro nanoparticles with incorporated SLC or CKR were pooled and concentrated as described above. A sample of fresh Salipro nanoparticles was kept and the rest was flash frozen in liquid nitrogen and stored at −80°C. Samples of fresh Salipro nanoparticles were compared to flash-frozen particles thawed on ice by analytical SEC as described above. In one experiment, *N*-linked glycans were removed from freeze-thawed Salipro-CKR nanoparticles by PNGase F treatment using the Glycoprofile II Enzymatic In-Solution *N*-Deglycosylation kit (Sigma-Aldrich) according to the manufacturer’s instructions. Briefly, 9 μL of protein or RNase B as positive control were incubated with 0.5 U of PNGase F at 37°C for 1 h, and analyzed by SDS-PAGE as described above.

### Negative-Stain EM

Three microliters of purified Salipro-SLC nanoparticles were applied on 400-mesh carbon-formvar coated copper grids (Ted Pella) and incubated for 30 s. Excess of sample was blotted off and grids were washed with ultrapure water prior to staining using 2% (w/v) uranyl acetate. Negative-stain image data were collected using a Hitachi HT7700 (Hitachi High-Technologies) transmission electron microscope operated at 120 kV and equipped with a 2K × 2K Veleta CCD camera (Olympus Soft Imaging Solutions).

### Western Blot Analysis

Proteins, originating from purified Salipro-CKR nanoparticles separated by SDS-PAGE, were transferred onto a polyvinylidene difluoride membrane (ThermoFisher Scientific) and blocked with 5% (w/v) BSA in Tris-buffered saline supplemented with 0.1% (v/v) Tween (TBST) at 4°C for 1 h. The membrane was incubated at room temperature and at 4°C for 1 h each on a rotator with a mouse monoclonal anti-Myc antibody directly conjugated to the fluorescent dye DyLight 680 (diluted 1:1000; ThermoFisher Scientific), and then washed with TBST. Fluorescence was detected in an iBright FL1000 imaging system (ThermoFisher Scientific).

## Results

### Direct Reconstitution Into Monodisperse Salipro-SLC Nanoparticles

**FIGURE 2:**
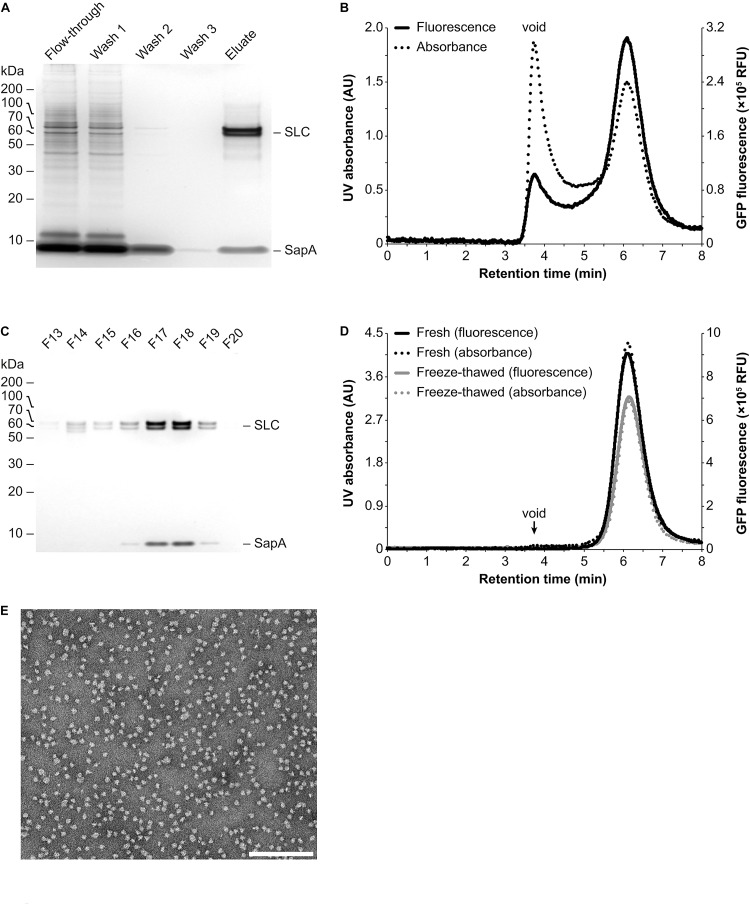
Purification and analysis of Salipro-SLC nanoparticles by chromatography. **(A)** Salipro nanoparticles containing SLC were separated by affinity chromatography from other Salipro-IMPs via the Strep-tag II affinity tag of SLC. Silver staining after SDS-PAGE of protein samples from the different purification steps showed that the eluate is pure as it contained only SLC and SapA. **(B)** Analytical SEC of the eluate from the affinity chromatography step with both UV (absorption at 280 nm) and fluorescence (excitation at 500 nm and emission at 512 nm) detection, able to monitor the daGFP reporter fused to SLC, revealed a homogenous population of Salipro-SLC nanoparticles. **(C)** The concentrated eluate from the affinity chromatography step was fractioned by preparative SEC. Fractions were visualized by silver staining after SDS-PAGE to verify the pure preparation of SLC in Salipro nanoparticles. **(D)** The pooled and concentrated SEC peak fractions [F17–19 in **(C)**] maintained their assembled state even after freeze-thawing as judged by analytical SEC. **(E)** Representative negative-stain electron micrograph showing a homogenous population of Salipro nanoparticles, which in this case each contain a single SLC. The scale bar represents 200 nm.

For initial proof-of-concept experiments, a human SLC transporter construct containing an N-terminal Strep-tag II affinity tag followed by a daGFP reporter was transiently expressed in HEK293 suspension cells (Canul-Tec et al., 2017). Homogenized crude cell membranes were prepared and subsequently incubated in the presence of the mild detergent digitonin (1% final concentration), which increases the membrane fluidity at 4°C, thus rendering lipids and IMPs more accessible for the following reconstitution step. In addition, detergents have been shown to activate SapA molecules from a closed into an open, lipid-binding conformational state at physiological pH (Chien et al., 2017). After removal of insoluble material by low speed centrifugation, the remaining material was incubated with an excess of SapA molecules to allow for the reconstitution of all IMPs, including their associated lipids, into Salipro nanoparticles (Figures 1A,B). Subsequently, affinity chromatography was performed in detergent-free buffer using the Strep-tag II affinity tag to isolate the Salipro-SLC nanoparticles from the background of all other Salipro-reconstituted IMPs (Figure 1C). Indeed, silver staining after SDS-PAGE of the different purification steps revealed a pure affinity chromatography eluate containing only SLC and SapA (Figure 2A). When a sample of the eluate was subjected to analytical SEC in detergent-free buffer, the profile exhibited a symmetric peak both with UV absorbance and fluorescence measurement of the daGFP reporter, confirming the formation of a homogeneous and monodisperse population of Salipro-SLC nanoparticles (Figure 2B). Salipro-SLC nanoparticles purified with affinity chromatography were then subjected to preparative SEC and peak fractions were collected, pooled and visualized by silver staining after SDS-PAGE, revealing a pure preparation of SLC and SapA (Figure 2C). In addition, freeze-thawed Salipro-SLC nanoparticles remain assembled as a homogeneous population as judged by analytical SEC (Figure 2D). Negative-stain EM further confirmed the presence of a homogeneous monodisperse population of single particles on the grid (Figure 2E). These results demonstrate that it is possible to directly reconstitute IMPs with SapA and the endogenous lipids present in a crude cell membrane preparation with the DirectMX workflow.

### Direct Reconstitution of a Wild-Type Human GPCR

**FIGURE 3:**
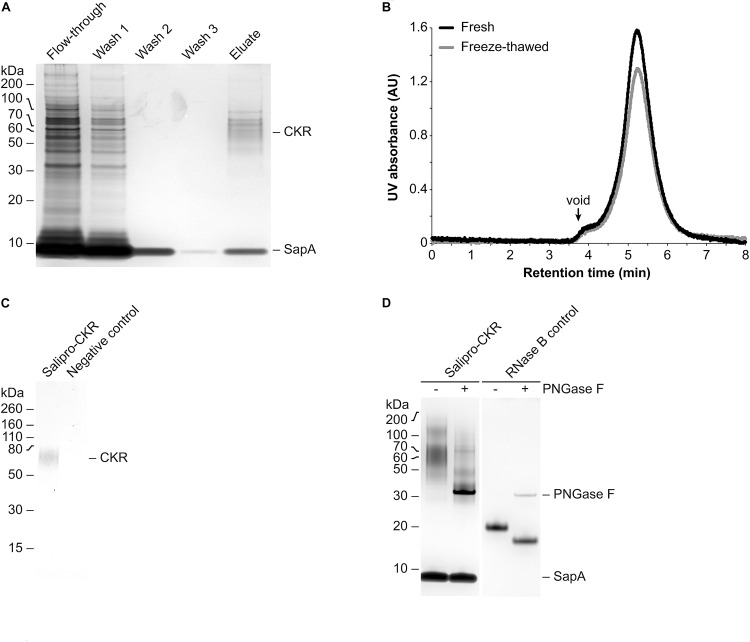
Purification and analysis of Salipro-CKR nanoparticles. **(A)** Salipro nanoparticles containing CKR were separated by affinity chromatography from other Salipro-IMPs via the DDK affinity tag of CKR. Silver staining after SDS-PAGE of samples from the different purification steps showed that the eluate contained protein bands corresponding to glycosylated CKR and SapA. **(B)** Peak fractions from the preparative SEC corresponding to Salipro-CKR nanoparticles were pooled and concentrated. The nanoparticles maintained their assembled state even after freeze-thawing as judged by analytical SEC with UV detection (absorption at 280 nm). **(C)** The presence of CKR in purified Salipro-CKR nanoparticles was verified by Western blots probed with a Myc-specific antibody. **(D)** De-glycosylation using PNGase F of purified Salipro-CKR nanoparticles resulted in reduced apparent molecular weights and “sharpened” protein bands as revealed by silver staining after SDS-PAGE. RNase B served as positive control.

To further demonstrate its potential, we applied the DirectMX methodology to a wild-type human CKR belonging to the GPCR superfamily, which represents one of the largest and most important protein classes for drug discovery. However, extraction and purification of wild-type GPCRs remains very difficult to achieve (for reviews see Hutchings et al., 2017; Hilger et al., 2018). Salipro-CKR nanoparticles were prepared using homogenized crude cell membranes from HEK293S suspension cells transiently expressing the wild-type human CKR fused to an N-terminal Myc-DDK dual affinity tag. Preparation of Salipro-CKR via the DirectMX workflow was essentially the same as presented above for SLC. Purification of Salipro-CKR nanoparticles was achieved by affinity chromatography, using the DDK affinity tag (Figure 3A), and subsequent preparative SEC. Pooled and concentrated SEC peak fractions remained homogenous and stable even after freeze-thawing as judged by the symmetric peak observed by analytical SEC (Figure 3B). Successful reconstitution of CKR into Salipro nanoparticles was further confirmed by Western blots probed with a Myc-specific antibody (Figure 3C). We hypothesized that Salipro-CKR nanoparticles are glycosylated since a “smeared” protein band was apparent by silver staining after SDS-PAGE (Figure 3D), despite the homogeneous peak from the analytical SEC step (Figure 3B). Indeed, it is known that the majority of all human CKRs carry 1–3 putative *N*-glycosylation sites, and the presence of *N*-linked glycan moieties have been experimentally demonstrated for several CKRs (reviewed by Szpakowska et al., 2012). Incubating the Salipro-CKR nanoparticles with PNGase F, an enzyme known to remove high mannose and complex *N*-linked glycans from glycoproteins, reduced the apparent molecular weights resulting in “sharpened” proteins bands, thus indicating the presence of *N*-linked glycans on the reconstituted CKR (Figure 3D). This example illustrates the potential of the Salipro technology to reconstitute a wild-type human GPCR solely using the endogenous lipids present in a crude cell membrane preparation by rapid affinity purification according to the DirectMX workflow.

## Discussion

Retaining IMPs in a lipid environment is essential to preserve both their structural and functional integrity (Whorton and MacKinnon, 2011; Contreras et al., 2012; Laganowsky et al., 2014; reviewed by Hedger and Sansom, 2016; Pyle et al., 2018). However, membrane proteins are most commonly purified in the continuous presence of detergents, leading to the partial or complete removal of the surrounding lipids (delipidation). This in turn often leads to protein aggregation and non-functional IMPs. IMP instability in detergent can be improved via protein engineering by mutagenesis (reviewed by Tate, 2012; Magnani et al., 2016). However, protein engineering is time-consuming and once a stable construct is obtained, it is fixed in a stable conformation, which prevents revealing the full structural and functional complexity of the native IMP. Furthermore, also protein-engineered IMPs need the continuous presence of detergents, which are known to have poor compatibility with many downstream analytic methods.

Several technologies have been developed to reconstitute IMPs into detergent-free environments. For example, detergent-purified IMPs can be reconstituted into a lipid environment using the nanodisc technology (reviewed by Denisov and Sligar, 2017). These nanoparticles are composed of small lipid bilayer disks encircled by a belt of fixed-size amphipathic MSPs derived from apolipoprotein A (Bayburt and Sligar, 2003). The limitation of a fixed nanodisc size requires screening multiple MSP constructs to find the suitable one that fits a certain IMP. In addition, optimizing the molar ratios between MSP, lipids, and IMP for each MSP construct may be time-consuming and resource-intensive. Most importantly, working with nanodiscs still requires a detergent-purified IMP as starting material, limiting its applicability when working with fragile targets.

Styrene-maleic acid copolymer-lipid particles represent an alternative system, in which the copolymer’s amphipathic nature is exploited for detergent-free extraction of small lipid bilayer disks containing single IMPs in their native lipid environment (Knowles et al., 2009; Lee et al., 2016). SMALPs have been used in several functional or structural studies on various IMPs (reviewed by Overduin and Esmaili, 2019). However, their broader applicability is limited due to unfavorable chemical properties of the styrene-maleic acid copolymer including its sensitivity to divalent cations, insolubility below pH 6.5 in aqueous solutions, and interference with UV absorption by proteins. Alternative copolymers, such as diisobutylene-maleic acid (Oluwole et al., 2017) or styrene-maleimide (Hall et al., 2018), have recently been developed to overcome the initial limitations of SMALPs, but in many cases their potential suitability to efficiently reconstitute IMPs from crude membranes remains to be validated (reviewed by Overduin and Esmaili, 2019). While both SMALPs and nanodiscs are valid and established methods, there still is no “one-solution-fits-all” approach when working with IMPs.

Recently, the Salipro technology has emerged as a novel IMP reconstitution tool. Here, the size-adaptable nature of the SapA scaffold allows for incorporation of both smaller and larger IMPs into Salipro nanoparticles by using the very same scaffold protein and workflow. The flexibility of the Salipro technology has been demonstrated by the reconstitution of a multitude of IMP sizes and shapes, ranging from small monomeric proteins, such as the 16.5-kDa bacterial outer membrane protein OmpX (Chien et al., 2017), to large protein oligomers, illustrated by the tetrameric peptide transporter PepT_*So*__2_ with a total of 56 transmembrane helices (Frauenfeld et al., 2016). The Salipro nanoparticles have been shown to be compatible with a wide range of high-resolution structural, biophysical and biochemical methods (Frauenfeld et al., 2016; Chien et al., 2017; Lyons et al., 2017; Flayhan et al., 2018; Kintzer et al., 2018; Nguyen et al., 2018; Kanonenberg et al., 2019; Kehlenbeck et al., 2019; Nagamura et al., 2019). Moreover, Salipro nanoparticles can be functionalized by a diverse range of tags on the SapA scaffold protein, useful for downstream applications relevant to pharmaceutical research. In addition, IMPs embedded in Salipro nanoparticles have been shown to be functional and can undergo conformational changes, highlighting the flexibility of the SapA scaffold to preserve the conformational changes required for protein function (Chien et al., 2017; Flayhan et al., 2018; Kanonenberg et al., 2019; Kehlenbeck et al., 2019).

Until now, the Salipro technology relied on detergent-purified IMPs, potentially limiting the possibility to work with unstable IMPs that are difficult to purify using standard approaches, as it is the case for many important human therapeutic targets (e.g., GPCRs, ion channels and transporters). To address this challenge, we have developed a novel Salipro reconstitution methodology termed DirectMX. The concept of DirectMX is to reconstitute IMPs directly from crude cell membranes, using the endogenous lipids present in crude cell membranes to enable subsequent detergent-free affinity purification of the native target protein.

DirectMX displays several features that facilitate the purification of challenging IMPs. First and foremost, DirectMX significantly shortens the time to obtain purified IMPs, as it obviates the need for laborious screening of suitable detergents. Detergents are not needed in the affinity purification step or for any downstream analysis. Moreover, it is not necessary to add artificial lipids since the IMP remains surrounded by its native membrane lipids, conserving important protein-lipid interactions that are important for structural and functional integrity. The entire workflow is straightforward and can be performed at 4°C, a feature especially important for fragile IMPs. This methodology has been shown to work for a broad range of IMPs from various expression systems (incl. human/insect cells and yeast).

In this proof-of-concept study, we present a novel Salipro methodology termed DirectMX. We illustrate the improved workflow by the reconstitution of a human SLC transporter and a wild-type human GPCR belonging to the CKR family. In both cases, the reconstituted protein is shown to form pure, stable and homogenous Salipro nanoparticles. Previously, detergent-purified IMPs reconstituted into Salipro nanoparticles were reported to remain structurally intact and functionally active (Chien et al., 2017; Flayhan et al., 2018; Kanonenberg et al., 2019; Kehlenbeck et al., 2019; Nagamura et al., 2019). We envision that the DirectMX methodology will enable studies on IMPs that remained so far inaccessible to other solubilization or reconstitution methods, in particular IMPs representing unstable therapeutic targets from the families of GPCRs, ion channels and transporters.

## Data Availability Statement

The datasets generated for this study are available on request to the corresponding author.

## Author Contributions

RL conceived and established the method. LC and MS selected CKR as target and provided CKR membranes. PL-G performed all experimental work with SLC and CKR. SK drafted the manuscript with input from RL, JF, and PL-G. All authors read, edited, commented on, and approved the final manuscript.

## Conflict of Interest

PL-G, SK, RL, and JF are employees of Salipro Biotech AB. LC and MS are employees of Bicycle Therapeutics Limited. DirectMX and the Salipro technology are protected i.a. by international patent applications as well as resulting national and regional phase patent family members.

## Abbreviations

CKR: chemokine receptor
CV: column volume
EM: electron microscopy
IMP: integral membrane protein
MSP: membrane scaffold protein
NMWL: nominal molecular weight limit
Salipro: saposin-lipoprotein
SapA: saposin A
SEC: size exclusion chromatography
SLC: solute carrier family 1 transporter
SMALP: Styrene-maleic acid copolymer-lipid particle.
